# Editorial: The future of oncology: digital twins and precision cancer care

**DOI:** 10.3389/frai.2026.1912241

**Published:** 2026-07-29

**Authors:** Eric Stahlberg, Jun Deng, Tina Hernandez-Boussard, Qi Wang, Hongfang Liu, Tanveer Syeda-Mahmood, Boris Aguilar, Huanmei Wu, Aidong Zhang

**Affiliations:** 1 Institute for Data Science in Oncology, University of Texas MD Anderson Cancer Center, Houston, TX, United States; 2 Department of Therapeutic Radiology, Yale University, New Haven, CT, United States; 3 Department of Biomedical Informatics and Data Science, Yale University, New Haven, CT, United States; 4 Department of Medicine, Stanford University, Stanford, CA, United States; 5 Department of Biomedical Data Science, Stanford University, Stanford, CA, United States; 6 Department of Mathematics, University of South Carolina, Columbia, SC, United States; 7 Department of Quantitative and Systems Health Sciences, University of Texas, Austin, TX, United States; 8 Institute for Systems Biology, Seattle, WA, United States; 9 Department of Health Services Administration and Policy, Temple University, Philadelphia, PA, United States; 10 Department of Computer Science, University of Virginia, Charlottesville, VA, United States

**Keywords:** artificial intelligence, clinical decision support, digital twin, *in silico* simulation, machine learning, multimodal data, multiscale modeling, precision oncology

The increasing availability and connectivity of computing and data resources are strengthening the environment for developing and successfully deploying “digital twin” approaches across various sectors. The digital twin approach, first employed by NASA and defined in a recent National Academies report (National Academies of Sciences, 2024), employs synchronized digital representations of individual real-world objects to help inform decisions involving that given object. This Research Topic explores the convergence of AI and digital twin approaches within oncology, a challenging dynamic biomedical space with hallmarks of tremendous complexity, high variability, limited data, and many difficult challenges to overcome.

With these challenges, one may ask why to pursue digital twin approaches for oncology at all? The answers are many. They are grounded in key aspects of the digital twin approach to observe, describe, apply, adapt, learn, and predict. This Research Topic brings together representative examples that embody these aspects, highlighting the key contributing role for AI in enabling a future where oncology digital twins are pervasive and benefiting patients globally. When the digital twin approach is applied broadly in oncology, the collective efforts enable insights to be learned from each oncology digital twin with the potential for use in new oncology digital twins to reduce the burden of cancer and improve the outcomes for cancer patients (Stahlberg et al., 2022).

The parallels among digital twin approaches for complex systems are found in the first paper, “*From data-driven cities to data-driven tumors: dynamic digital twins for adaptive oncology*” by Karaman and Sebin. The opportunities for digital twin approaches for oncology to improve cancer treatments are shared, as well as elements of success are also enumerated which include active involvement of the individual, acceptance by the clinician of the insights provided by the digital twin, and the need for longitudinal data from many individuals.

The papers “*DATA 5.0—Data Acquisition, Translation & Analysis—a prospective uro-oncological data warehouse for the 21st century*” by Schütz et al. and “*Understanding the need for digital twins' data in patient advocacy and forecasting oncology*” by Chang et al. delve into fundamental elements of the data underlying a successful digital twin in oncology. The collection, aggregation, and ethical sharing of longitudinal data for oncology digital twins are key pillars for the future of cancer patient digital twins. Ensuring data quality, harmonization, and representative sampling across diverse populations is critical, not only to realize the potential of more effective and cost-effective clinical trials, but also for ensuring the use of digital twins in oncology to improve outcomes across demographics. The role of AI to aid these efforts is also a key pillar to the future success for oncology digital twins, streamlining areas including collection, featurization, and selection of quality datasets. Furthermore, AI can provide data-driven models to help fill the gap when effective mechanistic approaches are unavailable.

The final two papers, “*Predictive digital twin for optimizing patient-specific radiotherapy regimens under uncertainty in high-grade gliomas*” by Chaudhuri et al., and “*Bridging the gap between hepatocellular carcinoma management guidelines and personalised medicine: a Bayesian network study*” by Wang et al., point the way forward with real examples of AI and digital twins in a clinical decision support capacity. While each is unique, both studies reinforce the fact that early-generation digital twins are most effective when engineered to inform on specific clinical questions. There are commonalities in the use of Bayesian methods to incorporate prior insights from both the individual patients and broader populations. The papers also highlight a key value proposition of cancer digital twins to incorporate the ever-expanding domain of patient data and observations in a common approach and framework, supporting the care team with new insights to guide increasingly optimal treatment decisions.

Digital twins in oncology research started to gain momentum after NCI and DOE jointly launched the Cancer Patient Digital Twin initiative in July 2020 (NCI-DOE, 2020). Observing across the papers in this Research Topic and the growing number of examples of cancer digital twins (Wu et al., 2022) and digital twins that have since been developed in other health areas (Kamel Boulos and Zhang, 2021), the pace of medical digital twin development is accelerating. Efforts such as the European Union supported Virtual Human Twin (European Commission, 2022), the Digital Twins for Health Society (2025), the Virtual Human Global Summit (Virtual Human Global Summit, 2025; Lin and Stahlberg, 2023), the Swedish Digital Twin Consortium (2019), and the Digital Twin Summit at MD Anderson Cancer Center all stand testament to the growing community involved in the development of medical digital twins.

A common theme emerging across these contributions is that the future success of oncology digital twins will depend as much on implementation, validation, and continuous improvement as on model development. While advances in AI, multimodal data integration, and computational modeling have accelerated the creation of increasingly sophisticated digital twins, demonstrating clinical utility and dynamical learning remains a critical next step. Future oncology digital twins must be evaluated not only for predictive accuracy but also for their ability to support clinical decision making, communicate uncertainty, integrate into existing workflows, and improve patient outcomes (Koul et al., 2025). Importantly, the most impactful digital twins may not be comprehensive virtual replicas of patients, but rather focused, continuously learning systems designed to support specific clinical decisions. As the field matures, rigorous validation, uncertainty quantification, and prospective evaluation will be essential to translating the promise of digital twins into routine oncology care.

As we look to the future for digital twins in oncology, there is tremendous opportunity to leverage the power of the medical digital twin community together with enabling technologies such as AI to transform cancer care. It will take true team data science efforts together with inspired innovations that challenge the status quo to make this paradigm shift (Hernandez-Boussard et al., 2021). Traversing this path will touch many stones along the way including increased levels of cooperation and trust, access to data, data engineering, models and insights, digital twin informed treatment pathways, new approaches to clinical trials, new computing infrastructure, increased community education, new levels of patient involvement, consistent global standardization, robust regulatory framework, sustainable funding, and extensive verification, validation, and uncertainty quantification (VVUQ) of AI models and digital twin frameworks (Deng, 2026).

This Research Topic serves to move us all further down this path, where cancer digital twins and AI work together to improve the outcomes for those touched by cancer.
